# Polyclonal emergence of virulent, multidrug-resistant *Edwardsiella tarda* lineages associated with bacillary necrosis of *Pangasius* in striped catfish farms

**DOI:** 10.1128/aem.02585-25

**Published:** 2026-06-11

**Authors:** Trong-Tuong Ho, Phung-Hieu Diep, Vinh Q. Tu, Khanh D. N. Ly, Nga P. Le, Oanh T. H. Dang, Andrew D. Millard, Tan-Trung Nguyen, Christian D. Nguyen, Binh An Diep, Thao P. H. Ngo, Cam T. Pham, Quoc B. Nguyen, Hoang A. Hoang

**Affiliations:** 1 Department of Biotechnology, Faculty of Chemical Engineering, Ho Chi Minh City University of Technology (HCMUT), Ho Chi Minh City, Vietnam; 2 Vietnam National University Ho Chi Minh City 54800, Ho Chi Minh City, Vietnam; 3 Evergreen Valley College 17068, San Jose, California, USA; 4 College of Aquaculture and Fisheries, Can Tho University, Can Tho, Vietnam; 5 Becky Mayer Centre for Phage Research, Division of Microbiology and Infection, University of Leicester 4488, Leicester, United Kingdom; 6 INSERM U1212 Acides nucléiques: Régulations Naturelle et Artificielle (ARNA), Institut Européen de Chimie et Biologie, Université de Bordeaux, Pessac, France; 7 Division of HIV, Infectious Diseases, and Global Medicine, Department of Medicine, University of California 166668, San Francisco, California, USA; 8 Aquacultural Biotechnology Division, Biotechnology Center of Ho Chi Minh City 736684 , Ho Chi Minh City, Vietnam; 9 Faculty of Biological Sciences, Nong Lam University 138176 , Ho Chi Minh City, Vietnam; Centers for Disease Control and Prevention, Atlanta, Georgia, USA

**Keywords:** *Edwardsiella tarda*, striped catfish aquaculture, genomic epidemiology, antimicrobial resistance, experimental infection model

## Abstract

Bacillary necrosis of Pangasius (BNP) is a recurrent bacterial disease that poses a major threat to Vietnam’s striped catfish (*Pangasianodon hypophthalmus*) aquaculture industry. Although BNP has traditionally been attributed to *Edwardsiella ictaluri*, its etiology remains unresolved due to limitations of species-specific diagnostics. In this study, we investigated acute BNP cases reported at 19 geographically distinct catfish farms across the Mekong River Delta from December 2023 to July 2024. While initial 16S rRNA PCR assays implicated *E. ictaluri*, whole-genome sequencing of isolates from 15 of 19 farms (79%) confirmed that all strains belonged to *Edwardsiella tarda*. Experimental challenge in a striped catfish infection model demonstrated that all 15 *E. tarda* strains were highly virulent, with reisolation of the pathogen fulfilling Koch’s postulates. Phylogenetic analysis identified six distinct clonal lineages, indicating a polyclonal population structure consistent with multiple introductions or diversification of endemic strains. Comparative genomics revealed conserved core genomes within lineages, with diversity in accessory regions driven by prophages and plasmids. Twelve prophages were identified across the genomes, clustering into conserved syntenic pairs and integrating at shared chromosomal hotspots. Antimicrobial susceptibility testing revealed multidrug-resistant phenotypes in multiple lineages, genetically linked to plasmid-borne resistance determinants. Plasmid analyses identified conjugative plasmids with conserved backbones that mediate horizontal transfer of multidrug resistance cassettes, including determinants conferring resistance to β-lactams, enrofloxacin, doxycycline, and trimethoprim-sulfamethoxazole. Collectively, these findings redefined *E. tarda* as the etiologic agent of recent BNP cases in Vietnam and revealed the genomic features underpinning its virulence and multidrug resistance.

**IMPORTANCE:**

Bacillary necrosis of Pangasius (BNP) is a serious disease affecting Vietnam’s striped catfish industry, one of the world’s largest freshwater aquaculture industries. For many years, BNP was attributed to *Edwardsiella ictaluri*, influencing how the disease was diagnosed and treated. In this study, we found that recent BNP outbreaks were instead caused by multiple strains of the related bacterium *Edwardsiella tarda*. Experimental infections confirmed that these strains can cause severe disease in striped catfish under controlled laboratory conditions. Some strains also carried multidrug resistance plasmids that were experimentally shown to transfer between bacteria, demonstrating a potential mechanism for the spread of antimicrobial resistance in aquaculture systems. We additionally developed a new PCR test that more accurately distinguishes *E. tarda* from closely related species. Together, these findings improve understanding of BNP and provide new tools for its disease diagnosis, surveillance, and management in aquaculture.

## INTRODUCTION

The striped catfish (*Pangasianodon hypophthalmus*) constitutes a major component of Vietnam’s aquaculture industry in the Mekong River Delta (1). The industry contributed 1.8–2.4 billion USD in exports, supplying 1.787 million tons of catfish to over 140 countries in 2024 (2). Despite its scale, production is periodically disrupted by bacterial diseases. One of the most important is bacillary necrosis of Pangasius (BNP), which causes recurrent and economically significant losses (1, 3). BNP has historically been attributed to *Edwardsiella ictaluri*, a gram-negative intracellular pathogen repeatedly isolated from diseased striped catfish in the Mekong Delta since the early 2000s (3–6).

The genus *Edwardsiella* includes *E. ictaluri*, *E. piscicida*, and *E. tarda*, all members of the *Enterobacterales* that can cause systemic infections in diverse aquatic and terrestrial hosts (7, 8). Despite its capacity to infect many different hosts, including catfish, tilapia, clown knifefish, turtles, crocodiles, frogs, toads, snakes, monkeys, and humans (8–14), *E. tarda* is regarded as an opportunistic pathogen that “warrants only minimal concern” to aquaculture (15). Accurate species-level identification is complicated by overlapping phenotypic traits and high sequence similarity in conserved loci such as 16S rRNA (7, 16). Consequently, diagnostics based solely on 16S rRNA amplification cannot reliably distinguish between *E. ictaluri* and *E. tarda* (17). However, emerging evidence suggests that *E. tarda* may play a previously unrecognized role in BNP outbreaks in the Mekong Delta (11).

To investigate the extent and potential etiologic agents associated with recent BNP cases, we examined diseased striped catfish reported by farm operators at 19 geographically distinct sites across the Mekong Delta region of Vietnam between December 2023 and July 2024. We combined genomic analyses, phylogenetics, virulence testing, and pangenome characterization of bacterial isolates recovered from these sites. Our findings address a critical gap in understanding *E. tarda* epidemiology in the Mekong Delta and lay the groundwork for improved diagnostics and surveillance.

## MATERIALS AND METHODS

### Study design

This study was designed as an integrated field, experimental, and genomic investigation to define the etiologic agent, population structure, virulence, and antimicrobial resistance determinants associated with BNP in striped catfish (*Pangasianodon hypophthalmus*) aquaculture systems in Vietnam. Between December 2023 and July 2024, acute mortality events consistent with BNP were reported at 19 geographically distinct striped catfish farms across the Mekong Delta. Clinically diseased fish from each site were examined grossly and subjected to bacteriological culture. Initial species identification was performed using phenotypic assays and *Edwardsiella ictaluri*-specific 16S rRNA PCR (17), reflecting diagnostic practices commonly used in regional aquaculture. One representative isolate from each of 15 farms yielding *Edwardsiella* spp. was selected for downstream analyses. Species-level identification and population structure were resolved using whole-genome sequencing, multilocus sequence typing (MLST), and phylogenetic comparison with a global *Edwardsiella* reference data set (18, 19). To overcome limitations of 16S rRNA-based diagnostics (17), a species-specific PCR assay targeting *Edwardsiella tarda* was developed and validated against closely related and commonly co-isolated species. Pathogenic potential of *E. tarda* isolates was assessed using a controlled experimental striped catfish infection model (20). Fish were challenged by immersion across multiple inoculum concentrations to evaluate dose-dependent mortality, estimate median lethal doses (LD_50_), and reproduce clinical and pathological features of acute BNP. Re-isolation and molecular confirmation of challenge strains were performed to satisfy Koch’s postulates. To characterize genomic features underlying lineage diversification and antimicrobial resistance, representative isolates from each MLST-defined clonal lineage underwent hybrid long- and short-read sequencing to generate closed genomes (21–27). Comparative analyses included core-genome single nucleotide polymorphism (SNP) phylogenetics, pangenome analysis (28, 29), prophage synteny analysis, and plasmid synteny analysis (30). Antimicrobial susceptibility testing using standardized MIC assays linked resistance phenotypes to plasmid-borne determinants. To directly evaluate the mobility of multidrug resistance plasmids inferred from genomic analyses, conjugation assays were performed using representative donor and recipient strains. These experiments assessed horizontal transfer of conjugative plasmids under laboratory conditions, with transconjugants validated by phenotypic antibiotic resistance profiles, MLST-based confirmation of recipient chromosomal background, and long-read whole-genome sequencing to verify plasmid acquisition.

See the supplemental material for detailed information on experimental procedures, bioinformatic workflows, primer sequences, antimicrobial susceptibility testing methods, and additional analyses.

## RESULTS

### Clinical presentation and geographic distribution of BNP-associated disease cases in the Mekong Delta

**Fig 1:**
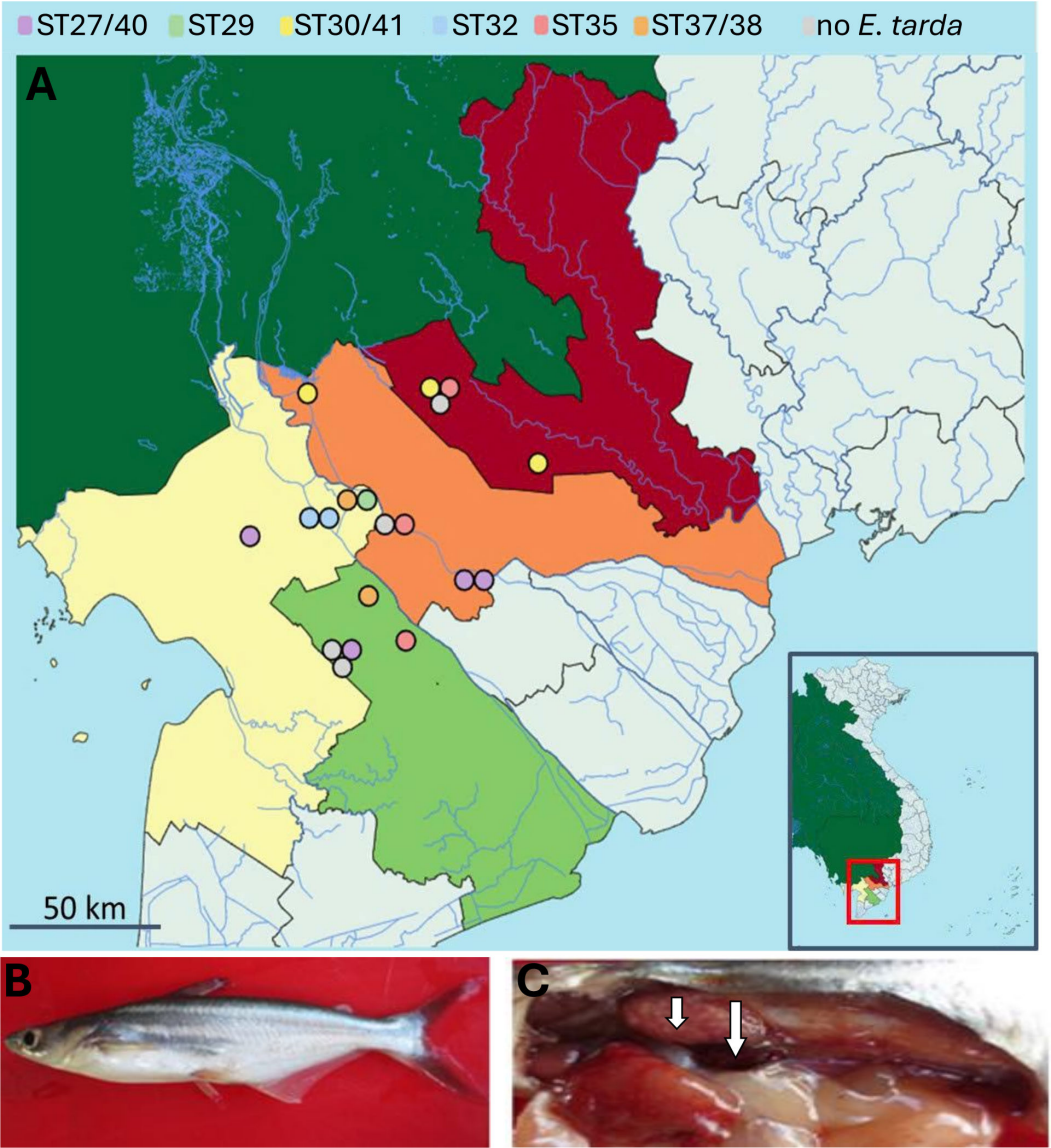
Geographic distribution and gross pathology of BNP-associated disease cases in striped catfish farms in the Mekong Delta. (**A**) Distribution of 19 striped catfish (*Pangasianodon hypophthalmus*) aquaculture farms from which BNP-associated disease cases were reported across four major production provinces of the Mekong Delta—An Giang, Cần Thơ, Đồng Tháp, and Tây Ninh—delineated according to Vietnam’s administrative consolidation effective July 2025 (Resolution No. 202/2025/QH15). Gray symbols indicate farms from which *Edwardsiella tarda* was not recovered, whereas colored symbols indicate farms from which *E. tarda* isolates were obtained, with colors corresponding to MLST-defined sequence types. The map was created using the Flourish Studio platform (Canva UK Operations Ltd.), with the underlying geographic boundary data sourced from the Open Development Vietnam portal (https://data.opendevelopmentmekong.net). (**B**) External appearance of a representative diseased fish, showing no specific external lesions, a presentation commonly observed in *Edwardsiella*-associated infections. (**C**) Internal gross pathology of a representative diseased fish, showing characteristic white nodules (white arrows) on the liver and spleen.

To characterize the geographic distribution and clinical features of BNP-associated disease cases in striped catfish, we examined acute mortality events reported by farm operators at 19 geographically distinct sites across the Mekong Delta region of Vietnam between December 2023 and July 2024. Affected sites were located across the four primary striped catfish-producing provinces of An Giang, Can Tho, Dong Thap, and Long An (Fig. 1A) (31). At each site, field investigators collected four to eight clinically diseased fish for diagnostic evaluation.

Affected fish commonly exhibited nonspecific clinical signs, including lethargy, surface swimming, anorexia, and diminished responsiveness to stimuli, which were initially presumed by farm operators to be consistent with *E. ictaluri* infection. Gross external examination of representative diseased fish revealed no overt cutaneous lesions (Fig. 1B). In contrast, internal examination consistently revealed prominent white nodules on the liver, kidney, and spleen (Fig. 1C), gross pathological features characteristic of BNP, a disease historically associated with *E. ictaluri* in the Mekong Delta since the early 2000s (3–6).

To investigate whether *E. ictaluri* was the causative agent of the observed disease, tissue samples from the liver, spleen, and kidney of diseased catfish were streaked onto *Edwardsiella ictaluri* agar (EIA), a selective medium commonly used for the isolation of *Edwardsiella* species. The resulting bacterial isolates were rod-shaped, gram-negative, weakly motile, catalase-positive, and capable of fermenting and oxidizing glucose, but negative for cytochrome oxidase activity, which are phenotypic traits consistent with *E. ictaluri*. PCR amplification using *E. ictaluri*-specific primers targeting a unique region of the 16S rRNA gene (17) produced a consistent 407-bp amplicon in isolates recovered from 15 of the 19 farms (79%), initially implicating *E. ictaluri* as the putative etiologic agent.

**Fig 2:**
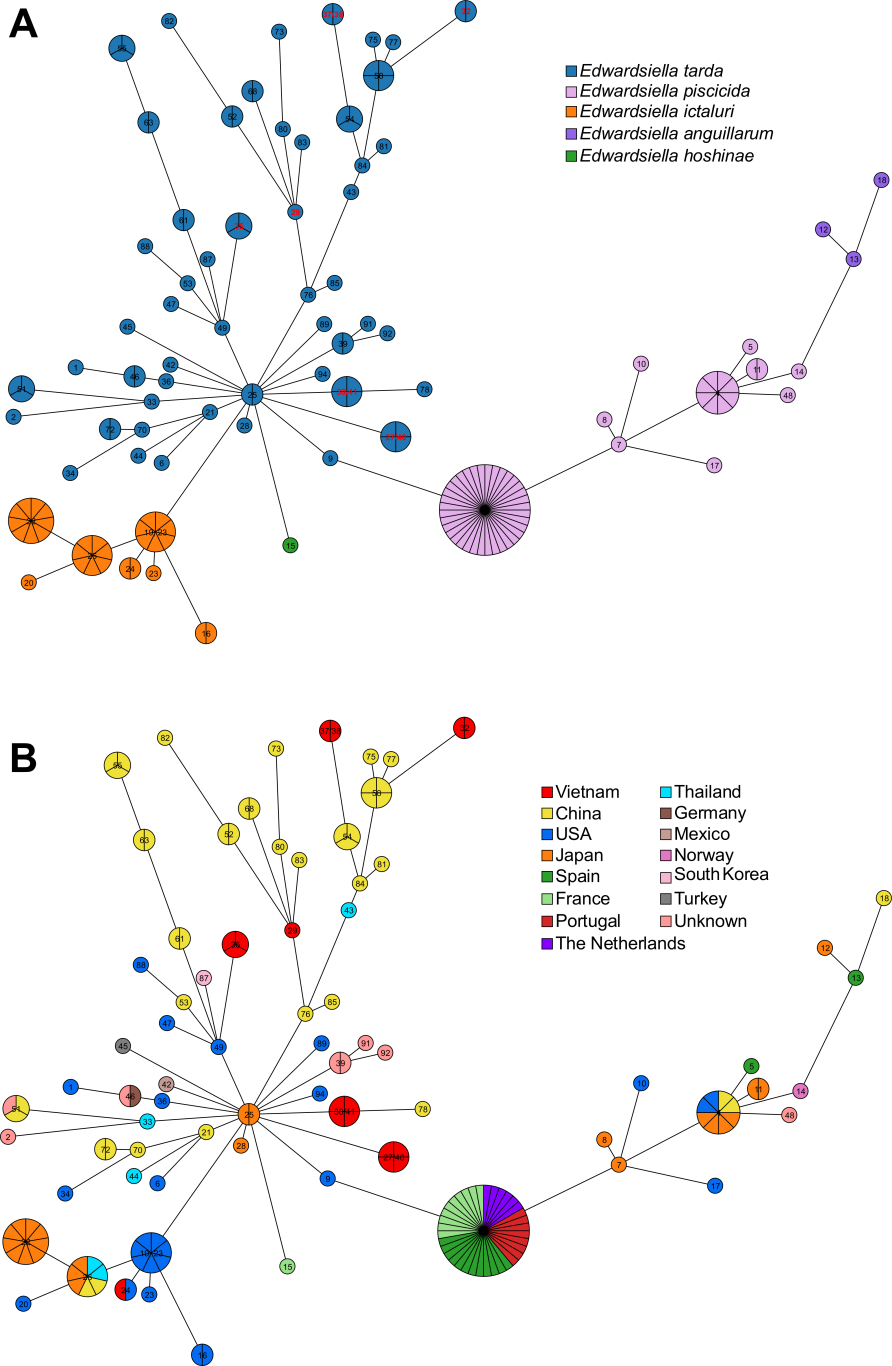
Minimum spanning tree of *Edwardsiella* spp. strains based on multilocus sequence typing. The tree was constructed using MLST allelic profiles from 10 housekeeping genes across 165 *Edwardsiella* isolates. Each node represents a unique sequence type, with the ST number displayed inside the node. Node size is proportional to the number of isolates sharing that ST. The lines connecting nodes indicate the number of allelic differences between sequence types, with shorter lines reflecting closer genetic relatedness. Nodes are color-coded by (**A**) species or (**B**) country of origin. In panel A, *E. tarda* strains from the present study are indicated by red node labels.

To further characterize the etiologic agents, one representative strain per farm (*n* = 15) was subjected to whole-genome sequencing using high-throughput Illumina technology. A minimum spanning tree was constructed from these draft genomes based on a 10-locus multilocus sequence typing (MLST) scheme (18, 19) (Fig. 2). For phylogenetic context, a global reference data set of 148 *Edwardsiella* isolates from 15 countries was retrieved from the public MLST database (18). Each node in the tree represents a unique sequence type (ST), with node size reflecting the number of isolates sharing that ST. When color-coded by species, the tree revealed clear species-level clustering, with *E. tarda*, *E. ictaluri*, *E. piscicida*, and *E. anguillarum* each occupying distinct regions of the tree (Fig. 2A). Notably, *E. ictaluri* clustered more closely with *E. tarda*, suggesting greater genetic similarity between these two species. *E. anguillarum* was more closely related to *E. piscicida*, indicating a separate evolutionary lineage. When color-coded by country of origin, all 15 strains from Vietnam clustered within the *E. tarda* clade (Fig. 2B), thereby identifying *E. tarda*—not *E. ictaluri*—as the etiologic agent. The earlier misidentification was due to high sequence similarity in the 16S rRNA region between *E. ictaluri* and *E. tarda*.

To overcome the limitations of 16S rRNA-based species identification (14), we designed a pair of *E. tarda*-specific primers (ETF/ETR) and evaluated their specificity against *E. tarda* and closely related or commonly co-isolated bacterial species. PCR amplification using the ETF/ETR primers produced the expected amplicon in representative *E. tarda* isolates from each MLST-defined clonal lineage, while no amplification was detected in *E. ictaluri*, *Aeromonas dhakensis*, or *Escherichia coli* strains (Fig. S1). These results confirm that the ETF/ETR primer pair provides species-specific detection of *E. tarda*, offering a reliable molecular tool for accurate discrimination from the closely related species *E. ictaluri*.

### Polyclonal distribution of *E. tarda* sequence types across the Mekong Delta

MLST analysis of *E. tarda* isolates recovered from diseased striped catfish revealed a polyclonal population structure across the Mekong Delta. Mapping of the 15 sampled farms showed their distribution across 11 districts within four major striped catfish-producing provinces, with districts representing administrative subdivisions of provinces (Fig. 1A). MLST resolved the isolates into six distinct STs (Table S1), demonstrating the presence of genetically diverse *E. tarda* lineages across sampled sites rather than dominance of a single clonal lineage.

Five STs were detected at multiple farms (Fig. 1A). For example, two ST32 isolates were recovered from farms within the Châu Thành District of An Giang Province, indicating spatial clustering of closely related strains at the district level. In contrast, ST27/40, ST30/41, ST35, and ST37/38 isolates were recovered from farms located in multiple provinces (Fig. 1A; Table S1). Together, these findings indicate that multiple genetically distinct *E. tarda* lineages were present across geographically separated farms in the Mekong Delta during the study period.

### Experimental infection of striped catfish demonstrates the pathogenic potential of *E. tarda* isolates

To evaluate the pathogenic potential of *E. tarda* isolates recovered from diseased striped catfish, we utilized an established experimental striped catfish infection model (20). One representative *E. tarda* isolate from each of the 15 farms was individually used to challenge healthy striped catfish at four inoculation doses (10^3^, 10^4^, 10^5^, and 10^6^ CFU per mL), with 60 fish per dose group, to assess their ability to reproduce clinical disease and gross pathological features consistent with acute BNP under controlled conditions. Sham-inoculated control fish experienced no mortality during the 14-day observation period. In contrast, fish challenged with any of the 15 *E. tarda* isolates developed rapidly lethal infections, with mortality occurring between days 1 and 8 post-infection (Fig. S2).

**Fig 3:**
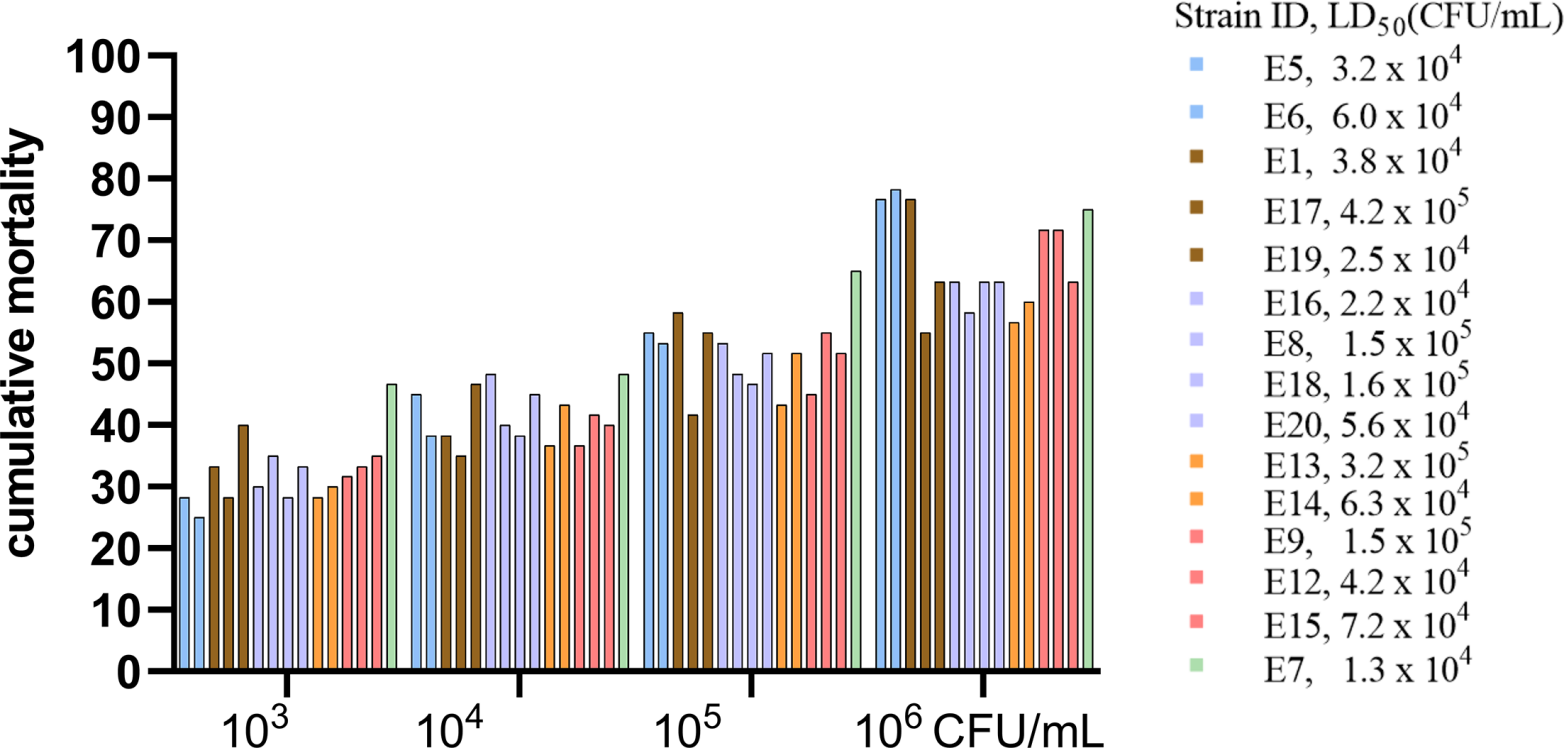
Experimental infection of striped catfish with *Edwardsiella tarda* isolates reproduces BNP-like disease under controlled conditions. Dose-dependent mortality of striped catfish (*Pangasianodon hypophthalmus*) following immersion challenge with 15 *E. tarda* isolates recovered from BNP-associated disease cases. Fish were exposed to four bacterial concentrations (10^3^, 10^4^, 10^5^, or 10^6^ CFU/mL; *n* = 60 fish per dose group), and survival was monitored for 14 days.

Survival analysis demonstrated a clear dose-dependent response, with higher bacterial concentrations resulting in more rapid and extensive mortality (Fig. S2). The median lethal dose (LD_50_), defined as the bacterial concentration required to induce mortality in 50% of infected fish, ranged between 10^4^ and 10^5^ CFU per mL for all 15 isolates, indicating comparable virulence across strains under experimental conditions (Fig. 3). Infecting bacteria were re-isolated from internal organs of diseased fish and exhibited colony morphology identical to the original challenge isolates on EIA. Re-isolated colonies were further confirmed by species-specific PCR, demonstrating that the experimentally introduced isolates were responsible for disease in the challenged hosts and satisfying the criteria of Koch’s postulates in this experimental infection model (32).

### Complete genome sequences of six *E. tarda* clonal lineages

**Fig 4:**
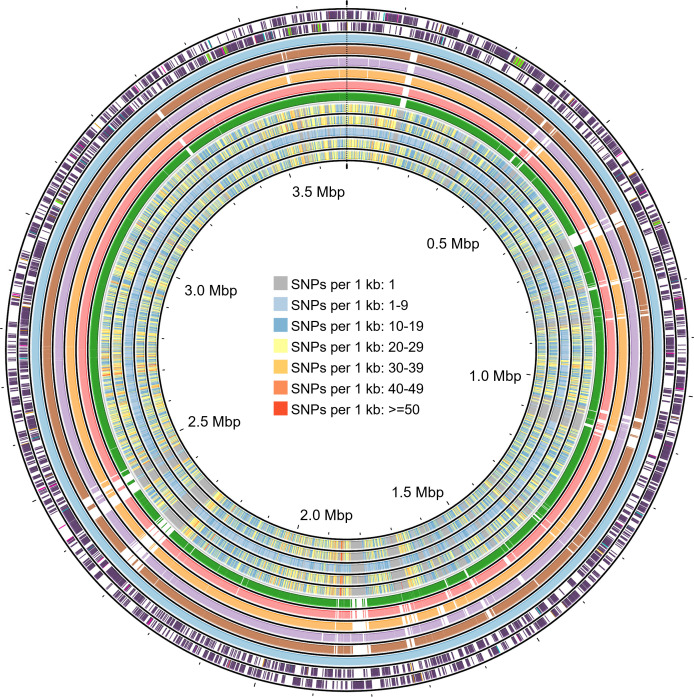
Circular genome atlas of six *E. tarda* isolates representing distinct clonal lineages. Each concentric circle, numbered from outermost circle to innermost circle, represents genomic data for the six *E. tarda* strains. Circles 1–2 represent predicted coding sequences on the plus and minus strands, respectively, for strain E24.5. Circles 3–8 show whole-chromosome alignments of the six strains relative to the E24.5 strain, specifically E24.5 (circle 3), E24.1 (circle 4), E24.16 (circle 5), E24.14 (circle 6), E24.9 (circle 7), and E24.7 (circle 8). Circles 9–13 display total SNPs per 1 kb relative to the E24.5 strain, specifically E24.1 (circle 9), E24.16 (circle 10), E24.14 (circle 11), E24.9 (circle 12), and E24.7 (circle 13).

To gain insight into the genomic features and potential virulence determinants of the 15 representative *E. tarda* isolates recovered from diseased striped catfish and characterized in the experimental catfish infection model, we used a hybrid sequencing approach combining PacBio long-read and Illumina short-read technologies to generate high-quality, closed genome assemblies for six representative strains corresponding to six distinct MLST-defined clonal lineages (Fig. 4). The chromosome sizes range from 3.46 to 3.85 Mbp, with G + C content varying slightly between 57.1% and 57.6% (Table S1). Each strain carries one to five plasmids that range in sizes from 3 to 152 kb (Table S3).

### Core-genome SNP phylogenetic analysis reveals polyclonal population structure of *E. tarda*

To further resolve the genetic relationships among *E. tarda* strains recovered from diseased striped catfish, we performed a core-genome SNP-based phylogenetic analysis using strain E24.5 as the reference (Fig. 4; Fig. S3). The resulting maximum-likelihood phylogeny resolved the 15 strains into six well-supported clades corresponding to MLST-defined clonal lineages, with strong bootstrap support across major nodes. Strains sharing the same sequence type exhibited minimal core-genome SNP divergence, with no SNP differences observed among ST32 and ST37/ST38 strains, 1 SNP among ST35 strains, 1–5 SNPs among ST27/40 strains, and up to 14 SNPs among ST30/41 strains, indicating high genomic conservation within clonal lineages. In contrast, strains from different sequence types differed by 25,667–63,102 core-genome SNPs (Fig. 4; Fig. S3). These findings indicate strong genomic conservation within lineages and substantial divergence between co-circulating *E. tarda* lineages.

### Comparative pangenome analysis reveals accessory gene variation among *E. tarda* isolates

**Fig 5:**
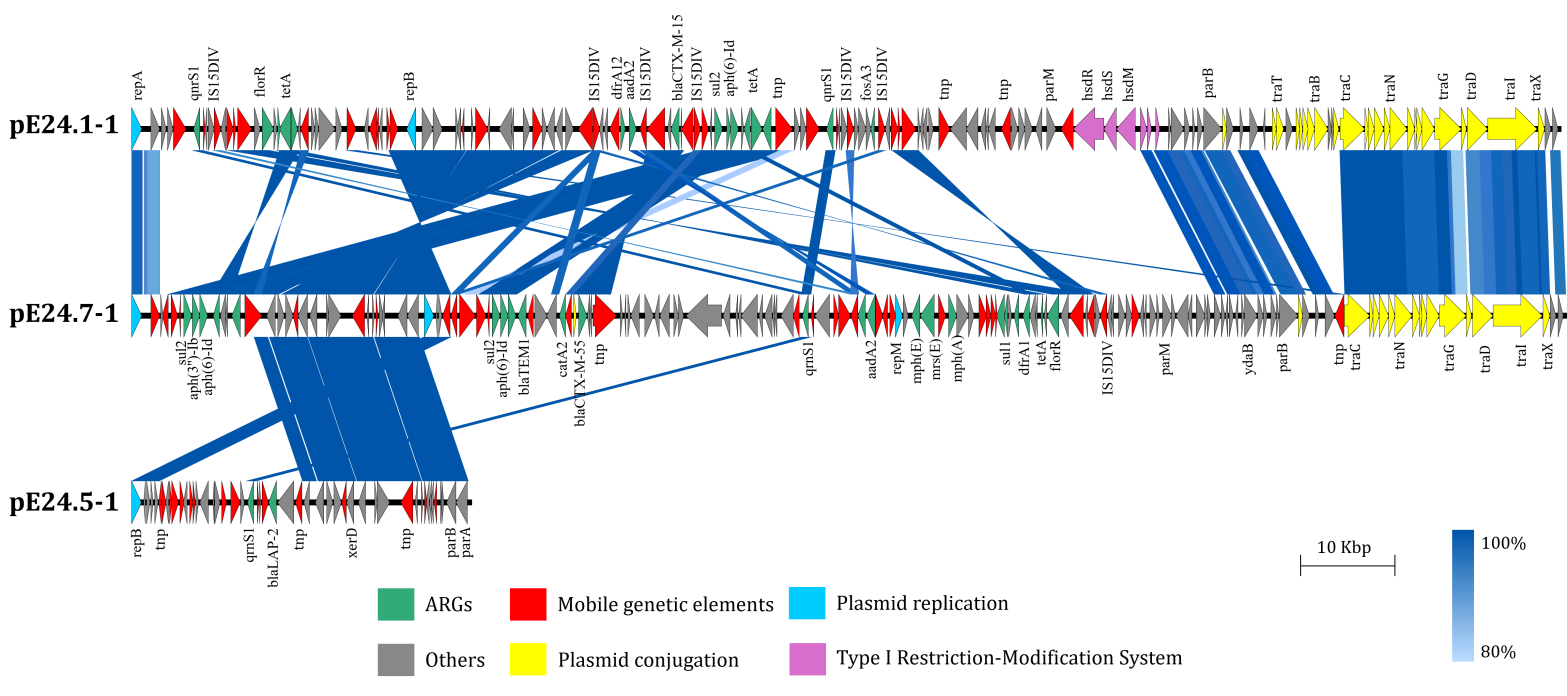
Synteny comparison of linearized plasmids from *E. tarda* strains. Coding sequences are represented as arrows and color-coded according to predicted gene function. Sequence similarity was assessed using the tBLASTx algorithm and is visualized by blue shading between plasmids, with intensity indicating similarity ranging from 80% to 100%.

To investigate genomic diversity beyond the core genome, we performed a pangenome analysis of 15 representative *E. tarda* isolates recovered from diseased striped catfish and visualized the results as a binary gene presence/absence matrix alongside a maximum-likelihood phylogenetic tree constructed from accessory gene content. While all strains shared a conserved core genome, the matrix revealed clear differences in accessory gene composition, highlighting accessory genes unique to each of the six identified *E. tarda* clonal lineages (Fig. S4). Notably, the structure of the phylogenetic tree constructed from accessory gene content (Fig. S4) was concordant with the MLST-based minimum spanning tree (Fig. 2) and genome-wide SNP-based phylogenetic tree (Fig. S3), reinforcing the resolution of the 15 strains into six genetically distinct clusters. The observed differences in accessory gene content among the 15 strains were primarily attributable to the horizontal acquisition of prophages (Fig. S5) and plasmids (Fig. 5).

### Prophages of *E. tarda* strains

A total of 12 complete prophages were identified across five of the six *E. tarda* clonal lineages, with no prophages detected in the ST30/41 lineage (Fig. S5). These 12 prophages were integrated at eight distinct chromosomal loci, including three conserved hotspots at 1.07, 1.70, and 1.80 Mbp, each harboring integration of two to three different prophages (Fig. 4). Genomic synteny analysis revealed considerable variation in prophage organization and sequence similarity (Fig. S5A). Eight of the prophages grouped into four syntenic pairs—E24.5-Φ2 with E24.16-Φ1, E24.5-Φ3 with E24.16-Φ3, E24.7-Φ1 with E24.14-Φ1, and E24.9-Φ1 with E24.16-Φ2—based on conserved gene order and high nucleotide identity (80%–100%). These syntenic pairs shared modular genome architectures, including genes for integrases, structural components, and lysis functions, with sequence divergence primarily confined to terminal regions encoding hypothetical proteins. Notably, three of the four syntenic pairs were integrated at shared chromosomal loci, highlighting conserved insertion hotspots targeted by closely related prophages (Fig. 4).

Phylogenetic analysis of the 12 *E. tarda* prophages alongside reference phages from the NCBI viral RefSeq database revealed that *E. tarda* prophages span multiple viral families and genera, based on intergenomic similarity threshold values recommended by the International Committee on Taxonomy of Viruses (Fig. S6) (33). Several clustered closely with known *Edwardsiella* phages, while others were affiliated with *Enterobacteria*, *Klebsiella*, or *Salmonella* phages (Fig. S5B). Notably, the four syntenic pairs identified in Fig. S5A also clustered together phylogenetically (Fig. S5B), supporting shared ancestry. In contrast, the four most divergent prophages—E24.5-Φ1, E24.5-Φ4, E24.7-Φ2, and E24.7-Φ3—lacked close relatives among reference sequences and formed distinct phylogenetic branches. These results highlight the contribution of diverse prophages to the diversification of the six *E. tarda* clonal lineages.

### Antimicrobial susceptibility patterns and associated resistance genes in *E. tarda* outbreak strains

ST29 and ST30/41 strains displayed a multidrug-resistant phenotype, exhibiting elevated MICs to β-lactams (cefixime), doxycycline, enrofloxacin, florfenicol, and trimethoprim-sulfamethoxazole; notably, ST29 strains also showed higher erythromycin MICs, whereas ST30/41 strains exhibited higher fosfomycin MICs (Table S2). In contrast, ST35, ST27/40, and ST37/38 strains generally exhibited lower MICs across most antimicrobial classes.

To define the genetic correlates of these resistance profiles, complete genome sequences were analyzed using the Comprehensive Antibiotic Resistance Database (34). For E24.1 and E24.7, which harbor large transposon-rich conjugative plasmids, we also performed long-read Oxford Nanopore Technologies (ONT) sequencing, yielding circularized plasmids pE24.1-1 (151,895 bp) and pE24.7-1 (152,451 bp) (Fig. 5; Table S3). Despite the presence of up to five plasmids in some outbreak strains (i.e., ST29 strain E24.7), antimicrobial resistance genes were clustered on large plasmids ranging from 36,188 to 152,451 bp (Table S3). In ST29 strain E24.7, the multidrug-resistant phenotype was associated with the presence of conjugative plasmid pE24.7-1 carrying multiple resistance genes, including β-lactamases (*CTX-M-55* and *TEM-1*), tetracycline resistance (*tetA*), sulfonamide resistance (*sul1*/*sul2*), trimethoprim resistance (*dfrA*), quinolone resistance (*qnrS*), and aminoglycoside resistance determinants [*aadA2*, *aph(3″)-Ib*, and *aph(6)-Id*] (Fig. 5; Table S3). Similarly, in ST30/41 strain E24.1, elevated MICs were associated with resistance genes encoded by plasmid pE24.1-1, carrying quinolone resistance (*qnrS1*), fosfomycin resistance (*fosA3*), β-lactamase (*CTX-M-15*), sulfonamide resistance (*sul2*), trimethoprim resistance (*dfrA*), and aminoglycoside resistance genes [*aadA2*, *aph(3″)-Ib*, and *aph(6)-Id*] (Fig. 5; Table S3). In ST35 strain E24.5, resistance genes were limited to pE24.5-1, carrying *LAP-2* and *qnrS1*; notably, LAP-2 does not confer resistance to third-generation cephalosporins such as cefixime (35) (Fig. 5; Tables S2 and S3).

Comparative synteny analysis further revealed two predominant resistance plasmid backbones among the *E. tarda* outbreak strains. Plasmids pE24.7-1 and pE24.1-1 shared syntenic regions encoding homologous conjugation-associated (*tra*) genes together with replication and partition functions, indicating a conserved conjugative backbone that has accumulated multiple distinct resistance gene cassettes flanked by transposase elements (Fig. 5). Plasmid pE24.1-1 encoded a Type I restriction–modification system (*hsdR–hsdS–hsdM*), a putative anti-restriction protein, and additional DNA methyltransferases, consistent with a defense-associated plasmid backbone (Fig. 5). Likewise, the non-conjugative plasmid pE24.5-1 shared syntenic regions encoding replication and partitioning functions, consistent with a conserved maintenance backbone that has similarly acquired distinct transposase-flanked resistance gene cassettes (Fig. 5).

### Conjugative transfer of pE24.1-1 confirmed by phenotypic and genomic analyses

**Fig 6:**
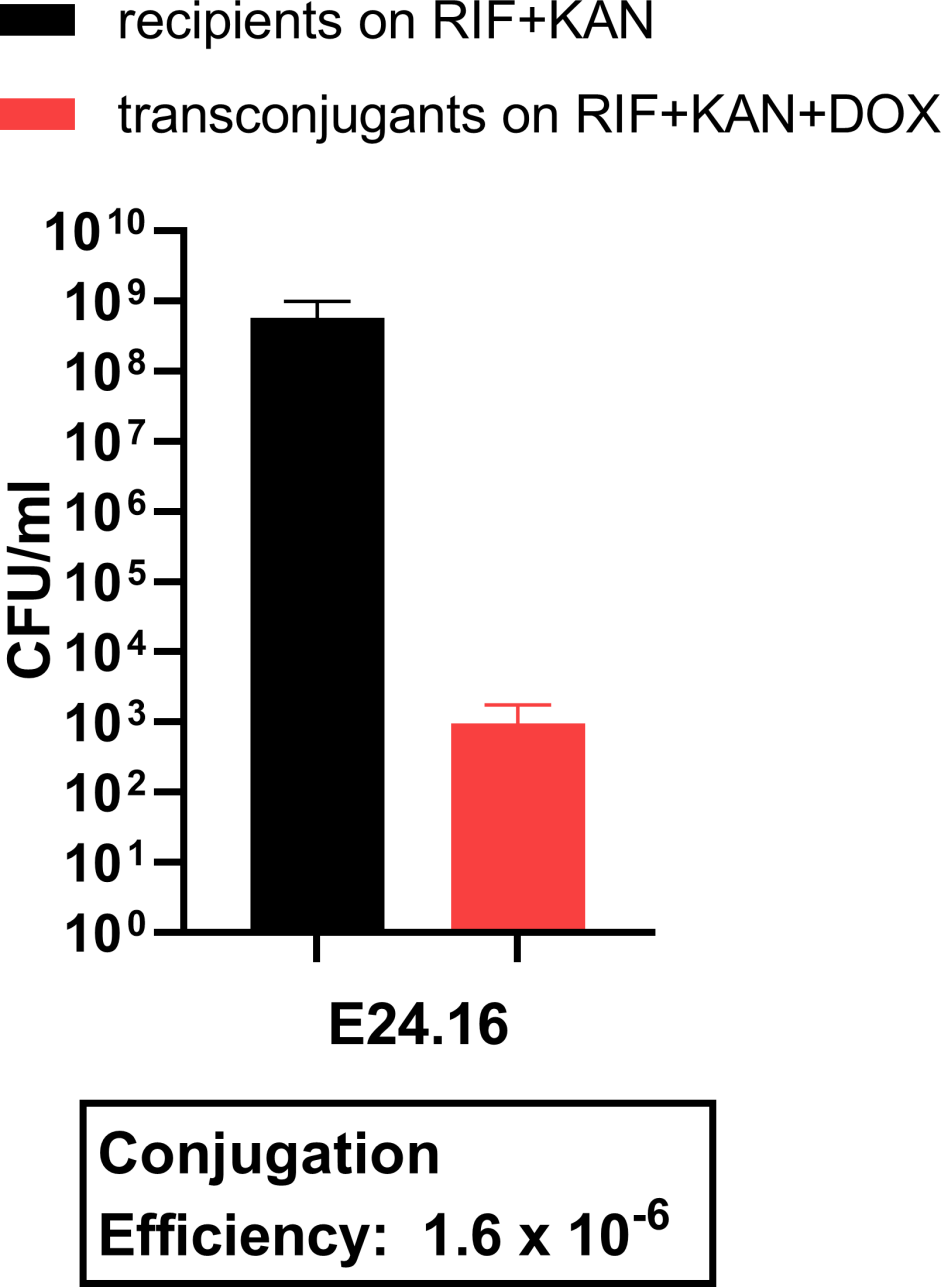
Conjugative transfer of pE24.1-1 from E24.1 to E24.16. Recipient cells of strain E24.16, marked with kanamycin and rifampicin resistance, were quantified on tryptic soy agar (TSA) supplemented with rifampicin (RIF) and kanamycin (KAN). Transconjugants, identified as recipient cells that acquired the *tetA*-harboring plasmid pE24.1-1, were selected and quantified on TSA supplemented with rifampicin, kanamycin, and doxycycline (DOX). A subset of transconjugants (n = 9), but not recipient cells lacking pE24.1-1, was further confirmed by replica plating for growth on TSA supplemented with cefixime, trimethoprim, or gentamicin. Bars represent CFU/mL recovered following filter mating. Error bars indicate standard deviation. Conjugation efficiency was estimated at 1.6 x 10^-6^ transconjugants per recipient.

To directly test whether the multidrug resistance plasmid in *E. tarda* is capable of horizontal transfer, we performed conjugation assays using two genetically unrelated strains: E24.1 as the donor and E24.16 as the recipient (Fig. 2; Table S3). The recipient strain E24.16 was sequentially marked by electroporation with the kanamycin resistance plasmid pCAT201 (36), which contains the broad-host-range pBBR1 replicon, and spontaneous rifampicin resistance to enable counterselection against the donor. Following filter mating, transconjugants were selected on tryptic soy agar (TSA) containing kanamycin, rifampicin, and doxycycline, the latter selecting for recipient cells that had acquired the tetracycline resistance determinant *tetA* encoded on the donor plasmid pE24.1-1 (Table S3), while excluding growth of both parental strains. Using this selection scheme, we recovered 9.5 × 10^2^ CFU exhibiting resistance to all three antibiotics (Fig. 6). In parallel, control plating on medium containing kanamycin and rifampicin yielded 5.8 × 10^8^ CFU, representing the total recipient population following filter mating. These data correspond to a conjugation efficiency of 1.6 × 10^−6^ transconjugants per recipient cell (Fig. 6).

To confirm the identity of the recovered colonies, nine randomly selected transconjugants were first assessed phenotypically. All nine isolates exhibited growth on TSA containing cefixime, trimethoprim, or gentamicin, consistent with acquisition of multiple resistance determinants associated with the donor plasmid pE24.1-1 (Fig. 6). To verify their genetic background, the same isolates were analyzed by sequencing the *gapA* locus used for MLST typing (19). All nine carried the *gapA*-16 allele, consistent with the chromosomal background of the recipient strain E24.16 (Table S1), thereby confirming that the recovered colonies were recipient-derived rather than donor carryover.

To further validate plasmid acquisition, one representative transconjugant was subjected to long-read Nanopore ONT whole-genome sequencing. Genome analysis confirmed an E24.16 chromosomal background and demonstrated the presence of the native small plasmid pE24.16-1, the introduced pCAT201 plasmid, and the conjugatively acquired pE24.1-1 plasmid, providing direct genomic evidence of plasmid transfer (GenBank accession number JBXXQG000000000). Taken together, these findings provide direct experimental evidence that the multidrug resistance plasmid pE24.1-1 identified in E24.1 is conjugative and capable of dissemination between *E. tarda* strains, supporting the genomic inference of plasmid-mediated spread of antimicrobial resistance observed in this study.

## DISCUSSION

Our investigation of BNP-associated disease cases reported from 19 striped catfish farms between late 2023 and mid-2024 revealed that *E. tarda* was frequently recovered from clinically diseased fish, accounting for isolates from 15 of 19 (79%) sampled farms (Fig. 1). Although initial diagnostics based on 16S rRNA PCR assays misidentified these isolates as *E. ictaluri*, whole-genome sequencing and MLST analysis confirmed that all 15 representative strains belonged to *E. tarda* (Fig. 2). This misclassification likely reflects the high sequence similarity between *E. tarda* and *E. ictaluri* within the 16S rRNA gene, underscoring the limited discriminatory power of 16S-based diagnostics for closely related *Edwardsiella* species.

The apparent absence of *Edwardsiella ictaluri* among recent BNP-associated disease cases in the Mekong Delta may reflect changes in epidemiology in combination with diagnostic limitations rather than true pathogen replacement. Long-term genomic surveillance in Vietnam has documented substantial temporal turnover in *E. ictaluri* lineages and accessory genome content over the past two decades, suggesting that currently circulating strains may differ from those historically associated with disease (6). In addition, reliance on culture-based workflows using *Edwardsiella ictaluri* agar—which also supports the growth of *E. tarda*—together with 16S rRNA-based diagnostics that may misclassify *E. tarda* as *E. ictaluri* (17), can reduce species-level resolution within the genus *Edwardsiella* and potentially contribute to underrecognition of *E. tarda* in prior BNP investigations.

Consistent with this interpretation, MLST and genome-wide SNP-based phylogenetic analyses revealed a polyclonal population structure among *E. tarda* isolates recovered from BNP-associated disease cases, with at least six genetically distinct lineages distributed across multiple provinces (Fig. 1 and 2; Fig. S3). The co-circulation of divergent sequence types argues against clonal expansion from a single source and instead supports a model of multiple introductions and/or diversification of endemic strains. Importantly, experimental infection of striped catfish confirmed that all 15 *E. tarda* strains were virulent and induced acute disease with comparable LD_50_ values, thereby fulfilling Koch’s postulates and establishing *E. tarda* as a bona fide BNP pathogen in this setting (Fig. 3; Fig. S2). These data challenge the long-standing perception of *E. tarda* as a primarily opportunistic pathogen of limited concern in aquaculture (15).

Comparative genomic analyses further showed that while core genomes were highly conserved within clonal lineages (Fig. S3), accessory genomes varied extensively (Fig. S4) and were shaped largely by prophages (Fig. S5) and plasmids (Fig. 5). Prophage analysis identified 12 complete prophages across five of six lineages, many of which formed syntenic and phylogenetic pairs integrated at conserved chromosomal loci (Fig. 5). This pattern suggests long-term association of specific prophages with *E. tarda* lineages and highlights the contribution of temperate phages to genome diversification.

A key finding of this study is the contribution of plasmids to antimicrobial resistance in *E. tarda*. Multidrug resistance phenotypes in ST29 and ST30/41 lineages were strongly associated with plasmid-borne resistance genes, including β-lactamases, quinolone resistance determinants, tetracyclines, sulfonamides, and aminoglycosides (Fig. S2 and S3). Synteny analysis revealed two plasmid backbones circulating among *E. tarda* strains. One represents a conserved conjugative backbone, shared by large plasmids, pE24.7-1 in ST29 and pE24.1-1 in ST30/41, encoding *tra* genes together with replication and partition functions and serving as a scaffold for the accumulation of multiple, distinct resistance gene cassettes flanked by transposase elements (Fig. 5). Consistent with these genomic predictions, conjugation assays demonstrated horizontal transfer of pE24.1-1 from strain E24.1 to E24.16, with phenotypic, MLST-based, and long-read sequencing confirmation of plasmid acquisition, providing direct experimental evidence that these multidrug resistance plasmids are capable of dissemination between *E. tarda* strains (Fig. 6). A second non-conjugative backbone, pE24.5-1 in ST32, encodes conserved replication and maintenance functions but carries lineage-specific resistance cassettes acquired through transposase-mediated rearrangements (Fig. 5). These observations support a model in which stable plasmid backbones act as repositories for resistance modules that can be independently acquired, reorganized, and maintained across lineages.

Limitations of this study include the selection of a single representative isolate from each of the 15 sampled striped catfish farms for whole-genome sequencing, which may underestimate within-farm genetic diversity or fail to capture mixed or co-infecting *Edwardsiella* strains. In addition, our analyses were restricted to culturable bacterial isolates recovered on *Edwardsiella ictaluri* agar and did not assess the potential contributions of other pathogens or broader microbial interactions to BNP-associated disease presentations. Because this study was not designed as a formal epidemiologic investigation, further longitudinal surveillance incorporating species-resolved diagnostics will be required to clarify the relative contributions of *E. tarda* and *E. ictaluri* to BNP in Vietnamese aquaculture systems.

In summary, our findings refine the current understanding of BNP-associated disease in Vietnam by demonstrating that a genetically diverse population of *Edwardsiella tarda* is frequently associated with diseased striped catfish and exhibits strong pathogenic potential under experimental conditions. These results have important implications for diagnostics, surveillance, and disease management in aquaculture systems. Diagnostic approaches capable of reliably distinguishing *E. tarda* from *E. ictaluri* are needed to improve species-level resolution, and future monitoring efforts would benefit from incorporating genome-based analyses to track the distribution and diversity of *E. tarda* lineages.

## SUPPLEMENTAL MATERIAL

Supplemental material aem.02585-25-s0001.docx Supplemental methods, Tables S1 to S3, and Fig. S1 to S6.

ASM does not own the copyrights to Supplemental Material that may be linked to, or accessed through, an article. The authors have granted ASM a non-exclusive, world-wide license to publish the Supplemental Material files. Please contact the corresponding author directly for reuse.
