# Supplementary material for: Polyclonal emergence of virulent, multidrug-resistant Edwardsiella tarda lineages associated with bacillary necrosis of Pangasius in striped catfish farms
Source: Appl Environ Microbiol. 2026 Jun 11;92(7):e02585-25. doi: 10.1128/aem.02585-25 (PMC13390456; doi:10.1128/aem.02585-25)
Supplement: Supplemental material — Supplemental methods, Tables S1 to S3, and Fig. S1 to S6. [file aem.02585-25-s0001.docx]

**SUPPLEMENTAL MATERIALS**

**METHODS**

**Characterization of *E. tarda* strains from diseased catfish.** Kidney and spleen samples were collected from clinically diseased striped catfish originating from 19 aquaculture farms in the Mekong Delta region of Vietnam and submitted for diagnostic evaluation. Sampling sites included Dong Thap Province (December 2023), Long An Province (December 2023 and July 2024), and Can Tho Province (June 2024). Bacterial isolation was performed by culturing samples on *Edwardsiella ictaluri* agar (EIA) at 28 °C for 24 to 48 hours. Isolates were preserved in tryptone soy broth (TSB; Merck) supplemented with 25% glycerol and stored at −80 °C.

**Experimental Striped Catfish Infection Model.** The experimental catfish infection model was approved by the Ethics Advisory Council on Animal Research of Can Tho University (CTU-AEC-3881/QD-DHCT). Juvenile striped catfish (*P. hypophthalmus*) weighing 7.5–10.0 g were obtained from a commercial hatchery and acclimated for 7 days in 500-L aerated plastic tanks. A total of 3,780 fish were used in three separate studies (E24.1, E24.5, E24.6 strains tested during March 2024; E24.7, E24.8, E24.9, E24.12, E24.13, E24.14, E24.15, E24.16 during June 2024; and E24.17, E24.18, E24.19, E24.20 during August 2024). Experimental infections were conducted as previously described (1) in 60-L plastic tanks, previously disinfected with 200 ppm chlorine, air-dried, and filled to two-thirds volume with municipal chlorinated water that was subsequently dechlorinated under continuous aeration prior to use.

*E. tarda* strains were recovered from archived stocks and streaked onto tryptone soy agar 1.5% (v/w) (TSA; Merck) for 36 h at 28°C. Pure colonies were confirmed via Gram staining and subcultured into tryptone soy broth (TSB) for 48 h at 28°C under shaking conditions. Bacterial densities were estimated spectrophotometrically at 610 nm and confirmed by serial dilution and colony enumeration on TSA. Inoculate were prepared to yield final challenge doses ranging from 10³ to 10⁶ CFU/mL.

For each of the three studies, fish were randomly assigned to five experimental groups (20 fish/group; 1 tank/group; n = 3 replicates per strain): (i) uninfected control, and (ii–v) four inoculum groups of 10³, 10⁴, 10⁵ and 10⁶ CFU/mL for each *E. tarda* strain. Fish were immersed in the corresponding bacterial suspension for 24 hours before transferring to clean, aerated tanks. Fish were monitored twice daily over a 14-day period for morbidity and mortality. Moribund and freshly dead fish were aseptically sampled (kidney) for bacterial re-isolation and confirmation by PCR. The median lethal dose (LD₅₀) was calculated based on cumulative mortality at each dose.

**PCR specificity assay.** The specificity of the *E. tarda*–specific primer pair ETF (5′-GTGTGCGTGTTAATAGCACCGTG-3′) and ETR (5′-AGGGTATCTAATCCTGTT TGCTC-3′) was evaluated against a panel of target and non-target bacterial species. Genomic DNA was extracted from bacterial cultures using the GeneJET Genomic DNA Purification Kit (Thermo Scientific) and used as a template for PCR amplification. Reactions were performed in a total volume of 25 µL containing PCR buffer, MgCl₂, dNTPs, primers (0.4 µM each), Taq DNA polymerase, and approximately 50 ng of template DNA. PCR amplification was carried out under the following cycling conditions: an initial denaturation at 95 °C for 5 min; followed by 30 cycles of denaturation at 95 °C for 30 s, annealing at 60 °C for 30 s, and extension at 72 °C for 45 s; with a final extension at 72 °C for 5 min. Amplification products were resolved by agarose gel electrophoresis and visualized under UV illumination after staining with GelRed fluorescent dye. Primer specificity was assessed based on the presence or absence of the expected amplicon in target versus non-target bacterial species.

**Whole Genome Sequencing.** Genomic DNA was extracted from 15 representative *E. tarda* strains using the GeneJET Genomic DNA Purification Kit (Thermo Scientific). All 15 genomic DNA samples were processed for high-throughput HackFlex library preparation and sequenced on the Illumina NovaSeq X platform (San Diego, CA, USA), generating 150 bp paired-end reads. Six of the 15 *E. tarda* isolates were additionally sequenced using the PacBio Revio platform (Menlo Park, CA, USA) with a 25M SMRT cell to obtain long-read data. Illumina and PacBio sequencing were performed by the DNA Technologies and Expression Analysis Cores at the University of California, Davis (Davis, California, USA). Two of the 15 *E. tarda* isolates, E24.1 and E24.7, harboring large transposon-rich conjugative plasmids were further sequenced using Oxford Nanopore Technologies (ONT) platform (Eurofins Genomics, Louisville, Kentucky) to resolve plasmid structure, yielding complete circularized plasmids pE24.1-1 and pE24.7-1. The sequenced genomes were deposited in GenBank under BioProject accession number PRJNA1345928.

**Bioinformatic analysis.** For the nine *E. tarda* isolates with Illumina short reads only, the reads were quality-filtered and adapter-trimmed using Trim Galore v0.6.10 (2). De novo assembly was performed using Unicycler v0.4.8 (3) with default settings, and the resulting assemblies were polished using Pilon v1.24 (4) to correct base-level errors and small misassemblies. Genome coverage was calculated as the total number of quality-filtered bases divided by the final assembly size, yielding an average short read coverage depth of 600–1000x.

For the six *E. tarda* isolates with long-read data, PacBio reads were downsampled and quality-filtered using Filtlong v0.2.1 (5) to a target of 1 Gb, producing ~50,000 high-quality reads with average length of ~20kb. Hybrid assemblies were generated with Unicycler v0.4.8, integrating both Illumina short- and PacBio long-read data, and polished two rounds with Racon v1.5.0 (6) using long reads and one round with Pilon v1.24 using normalized Illumina reads as implemented in the BV-BRC pipeline using default parameters (7). Post-assembly filtering of contigs was performed using Bowtie2 v2.4.4 (8) and Minimap2 v2.24-r1122 (9) to remove spurious or low-confidence sequences. Assembly graphs were visualized using Bandage v0.8.1, and quality metrics were assessed with QUAST v5.2.0.

**Multi-locus sequence typing (MLST) analysis.** MLST was performed using the PubMLST database (10, 11) for six complete and nine draft genome sequences. Among the ten MLST housekeeping genes for *Edwardsiella* spp., two copies of the *tuf* gene are present in each genome. For draft genome sequences missing one *tuf* allele, guided recovery was done using a modified BacTag pipeline (12). Paired-end reads were aligned to a 10 kb reference containing the missing allele using BWA-MEM, sorted with SAMtools, and per-base coverage calculated.

**Comparative Genomic Analysis.** Complete and draft genome assemblies of 1**5** *E. tarda* isolates were analyzed using Proksee (13). Draft genomes were aligned against a complete reference genome using Proksee’s integrated BLAST-based comparison tool. Predicted prophage regions, identified using the PHASTEST tool (14), are overlaid on the maps for structural context.

Whole-genome single-nucleotide polymorphism (SNP) analysis was performed to infer phylogenetic relationships among *E. tarda* strains relative to the reference strain *Edwardsiella tarda* E24.5. SNP calling was conducted using Snippy by aligning draft genome assemblies to the E24.5 reference genome. A core genome SNP alignment was generated with Snippy-core, which removed invariant positions and filtered complex variants to retain only orthologous SNP sites shared across all genomes. Maximum-likelihood phylogenetic reconstruction was performed using IQ-TREE under TPM2u+F model, with 1,000 bootstrap replicates. The resulting phylogeny was visualized and annotated using iTOL.

The pan-genome of the isolates was determined using the Roary pipeline (v.3.12.0) (15) on the set of annotated *E. tarda* genomes. Genes were clustered with a minimum BlastP identity of 95%, and the core genome was defined as genes present in at least 95% of the isolates. The resulting pan-genome distribution and the reconstructed phylogenetic tree were visualized and analyzed using packages within the ComplexHeatmap framework (16) in the Rstudio.

**Antimicrobial Susceptibility Testing.** Minimum inhibitory concentrations (MICs) were determined for *E. tarda* isolates using the broth microdilution method in accordance with Clinical and Laboratory Standards Institute guideline (17). Custom 96-well microdilution panels (Thermo Fisher Scientific) contained twofold serial dilutions of the following antimicrobial agents: cefixime (CEF), colistin (COL), doxycycline (DOX), enrofloxacin (ENR), erythromycin (ERY), gentamicin (GEN), kanamycin (KAN), florfenicol (FFC), fosfomycin (FOS), trimethoprim (TMP), and sulfamethoxazole (SMX). Quality-control strain *Escherichia coli* ATCC 25922 was tested in parallel to confirm panel performance.

**Conjugation Assay.** Conjugation assays were performed to assess horizontal transfer of the multidrug resistance plasmid pE24.1-1 from *E. tarda* strain E24.1 (donor) to strain E24.16 (recipient). The recipient strain E24.16 was first marked by electroporation (18) with plasmid pCAT201 (26), which carries a kanamycin resistance determinant and the broad-host-range pBBR1 origin of replication. To enable double counterselection against the donor strain, a rifampin-resistant derivative of E24.16/pCAT201 was subsequently selected by plating on tryptic soy agar (TSA) supplemented with kanamycin (50 µg/mL) and rifampin (50 µg/mL).

For conjugation assay by filter mating, overnight cultures of donor and recipient strains were grown in tryptic soy broth (TSB) and mixed at a 1:4 (recipient:donor) ratio. Log-phase cell mixtures were collected by centrifugation, resuspended in a small volume of TSB, and applied onto sterile 0.45-µm membrane filters (EMD Millipore, catalog number: HAWP02500) placed on TSA plates. Following incubation at 30°C for 20 h, cells were recovered from filters by resuspension in sterile phosphate-buffered saline and serially diluted.

Transconjugants were selected on TSA supplemented with kanamycin (50 µg/mL), rifampin (50 µg/mL), and doxycycline (2 µg/mL), the latter selecting for acquisition of the *tetA*-harboring plasmid pE24.1-1. Recipient cells were enumerated on TSA containing kanamycin and rifampin only. Colonies were counted after incubation at 30°C for 24 h. Conjugation efficiency was calculated as the number of transconjugant CFU divided by the number of recipient CFU.

To confirm acquisition of plasmid-associated resistance phenotypes, a subset of transconjugants (n = 9) was subjected to replica plating onto TSA supplemented individually with cefixime (5 µg/mL), trimethoprim (25 µg/mL), and gentamicin (10 µg/mL). Growth on all selective media was interpreted as evidence of acquisition of multidrug resistance determinants associated with pE24.1-1.

To confirm the identity of transconjugants, nine colonies were randomly selected for sequencing of the *gapA* locus used for MLST using the primers, Et-gapA-F (5’-CWACTCACGGTCGTTTCAAC-3’) and Et-gapA-R (5’-CGTTGTCATACCAAGAAACC), redesigned to better match the *E. tarda* chromosome. The 750-bp amplicons were generated by PCR and analyzed by Sanger sequencing (UC Berkeley DNA Sequencing Facility, Berkeley, California) and allele assignments were determined by comparison to the *Edwardisella* MLST database (10, 11).

For genomic confirmation, one representative transconjugant was subjected to long-read sequencing using the Oxford Nanopore Technologies (ONT) platform (Eurofins Genomics, Louisville, Kentucky). Reads were assembled using long-read assembly pipelines, and resulting contigs were analyzed to confirm chromosomal background and plasmid content using Proksee’s integrated BLAST-based comparison tool (13).

**Supplemental Table 1.** Genomic characteristics and multilocus sequence typing (MLST) of *E. tarda* strains.

| Strain | Province | District* | Size (Mbp) | Complete level | %GC | CDS (total) | ST | *adk* | *atpD* | *dnaJ* | *gapA* | *glnA* | *hsp60* | *phoR* | *pyrG* | *rpoA* | *tuf* |
| --- | --- | --- | --- | --- | --- | --- | --- | --- | --- | --- | --- | --- | --- | --- | --- | --- | --- |
| E24.7 | An Giang | Chợ Mới | 3.85 | Complete | 56.9 | 3443 | ST29 | 13 | 1 | 1 | 19 | 15 | 5 | 18 | 20 | 1 | 17,17 |
| E24.1 | Đồng Tháp | Hồng Ngự | 3.69 | Complete | 57.2 | 3243 | ST30/41 | 11 | 1 | 1 | 17 | 11 | 5 | 16 | 8 | 1 | 14,17 |
| E24.19 | Tây Ninh | Tân Hưng | 3.68 | Contigs | 56.9 | 3234 | ST30/41 | 11 | 1 | 1 | 17 | 11 | 5 | 16 | 8 | 1 | 14,17 |
| E24.17 | Tây Ninh | Tân Thạnh | 3.62 | Contigs | 57.3 | 3209 | ST30/41 | 11 | 1 | 1 | 17 | 11 | 5 | 16 | 8 | 1 | 14,17 |
| E24.5 | An Giang | Châu Thành | 3.72 | Complete | 57.3 | 3303 | ST32 | 12 | 12 | 14 | 18 | 14 | 14 | 17 | 19 | 8 | 16,16 |
| E24.6 | An Giang | Châu Thành | 3.67 | Contigs | 57.3 | 3278 | ST32 | 12 | 12 | 14 | 18 | 14 | 14 | 17 | 19 | 8 | 16,16 |
| E24.14 | Cần Thơ | Cờ Đỏ | 3.53 | Complete | 57.6 | 3070 | ST37/38 | 12 | 13 | 16 | 21 | 17 | 16 | 20 | 22 | 8 | 2, 16 |
| E24.13 | An Giang | Chợ Mới | 3.46 | Contigs | 57.6 | 3039 | ST37/38 | 12 | 13 | 16 | 21 | 17 | 16 | 20 | 22 | 8 | 2, 16 |
| E24.9 | Cần Thơ | Ô Môn | 3.59 | Complete | 57.4 | 3122 | ST35 | 14 | 1 | 15 | 20 | 16 | 15 | 19 | 21 | 1 | 17,17 |
| E24.12 | Đồng Tháp | Cao Lãnh City | 3.50 | Contigs | 57.5 | 3091 | ST35 | 14 | 1 | 15 | 20 | 16 | 15 | 19 | 21 | 1 | 17,17 |
| E24.15 | Tây Ninh | Tân Hưng | 3.50 | Contigs | 57.5 | 3091 | ST35 | 14 | 1 | 15 | 20 | 16 | 15 | 19 | 21 | 1 | 17,17 |
| E24.16 | Đồng Tháp | Châu Thành | 3.69 | Complete | 57.1 | 3272 | ST27/40 | 1 | 11 | 13 | 16 | 13 | 13 | 15 | 18 | 2 | 13,17 |
| E24.18 | Cần Thơ | Thới Lai | 3.63 | Contigs | 57.2 | 3252 | ST27/40 | 1 | 11 | 13 | 16 | 13 | 13 | 15 | 18 | 2 | 13,17 |
| E24.8 | An Giang | Châu Thành | 3.63 | Contigs | 57.2 | 3254 | ST27/40 | 1 | 11 | 13 | 16 | 13 | 13 | 15 | 18 | 2 | 13,17 |
| E24.20 | Đồng Tháp | Châu Thành | 3.63 | Contigs | 57.2 | 3250 | ST27/40 | 1 | 11 | 13 | 16 | 13 | 13 | 15 | 18 | 2 | 13,17 |

*Châu Thành District in An Giang Province and Châu Thành District in Đồng Tháp Province are distinct geographic locations, despite sharing the same district name.

**Supplemental Table 2.** Minimum inhibitory concentrations against 11 antimicrobial agents for *E. tarda* strains.*

| Strain | ST | CEF | DOX | ENR | ERY | GEN | KAN | FFC | FOS | TMP | SMX | COL |
| --- | --- | --- | --- | --- | --- | --- | --- | --- | --- | --- | --- | --- |
| E24.7 | ST29 | 64 | 32 | 16 | 64 | 4 | 8 | 64 | 0.5 | 64 | >512 | 64 |
| E24.1 | ST30/41 | 64 | 16 | 16 | 16 | 2 | 16 | 64 | 16 | 64 | >512 | 64 |
| E24.19 | ST30/41 | 64 | 8 | 16 | 16 | 2 | 4 | 64 | 16 | 64 | >512 | 64 |
| E24.17 | ST30/41 | 64 | 4 | 16 | 8 | 1 | 8 | 64 | 16 | 64 | >512 | 64 |
| E24.5 | ST32 | 0.125 | 0.25 | 0.25 | 16 | 1 | 8 | 0.5 | 1 | 0.125 | 32 | 32 |
| E24.6 | ST32 | 0.25 | 0.25 | 0.25 | 16 | 2 | 8 | 0.125 | 2 | 0.125 | >512 | 64 |
| E24.14 | ST37/38 | 0.125 | 0.25 | 0.125 | 8 | 2 | 8 | 1 | 1 | 0.125 | 128 | 32 |
| E24.13 | ST37/38 | 0.125 | 0.25 | 0.125 | 8 | 2 | 8 | 1 | 1 | 0.125 | 128 | 32 |
| E24.12 | ST35 | 0.125 | 0.125 | 0.125 | 16 | 2 | 4 | 1 | 1 | 0.125 | 16 | 64 |
| E24.15 | ST35 | 0.5 | 0.25 | 0.125 | 8 | 2 | 4 | 1 | 2 | 0.125 | 128 | 64 |
| E24.16 | ST27/40 | 0.5 | 0.25 | 0.125 | 8 | 2 | 4 | 1 | 0.5 | 0.125 | 256 | 32 |
| E24.18 | ST27/40 | 0.25 | 0.25 | 0.125 | 8 | 2 | 4 | 1 | 0.5 | 0.125 | 128 | 16 |
| E24.8 | ST27/40 | 0.125 | 0.25 | 0.125 | 8 | 2 | 8 | 1 | 0.5 | 0.125 | 128 | 32 |
| E24.20 | ST27/40 | 0.25 | 0.25 | 0.125 | 16 | 2 | 8 | 1 | 0.5 | 0.125 | 128 | 16 |

* MIC values are reported in µg/mL for CEF, cefixime; COL, colistin; DOX, doxycycline; ENR, enrofloxacin; ERY, erythromycin; GEN, gentamicin; KAN, kanamycin; FFC, florfenicol; FOS, fosfomycin; TMP, trimethoprim; SMX, sulfamethoxazole.

**Supplemental Table 3.** Plasmid-associated antimicrobial resistance genes identified by CARD analysis in *E. tarda* strains.

| **Strain** | **Plasmid** | **Size (bp)** | **Resistance genes** |
| --- | --- | --- | --- |
| E24.7 | pE24.7-1 | 152,451 | *CTX-M-55, TEM-1, tetA, sul1, sul2, dfrA1, qnrS, qacEdelta1, floR, aph(3'')-Ib, aph(6)-Id* |
|  | pE24.7-2 | 6,544 |  |
|  | pE24.7-3 | 4,758 |  |
|  | pE24.7-4 | 2,883 |  |
| E24.1 | pE24.1-1 | 151,895 | *CTX-M-15, blaCARB-2, tetA, fosA3, sul2, dfrA12, qnrS1, aadA2, aph(3″)-Ib, aph(6)-Id* |
|  | pE24.1-2 | 7,172 |  |
|  | pE24.1-3 | 5,298 |  |
|  | pE24.1-4 | 2,888 |  |
|  | pE24.1-5 | 2,773 |  |
| E24.5 | pE24.5-1 | 36,188 | *LAP-2, qnrS1* |
|  | pE24.5-2 | 2,969 |  |
|  | pE24.5-3 | 2,888 |  |
| E24.14 | pE24.14-1 | 6,386 |  |
|  | pE24.14-2 | 6,162 |  |
|  | pE24.14-3 | 4,678 |  |
| E24.9 | pE24.9-1 | 3,039 |  |
| E24.16 | pE24.16-1 | 3,780 |  |


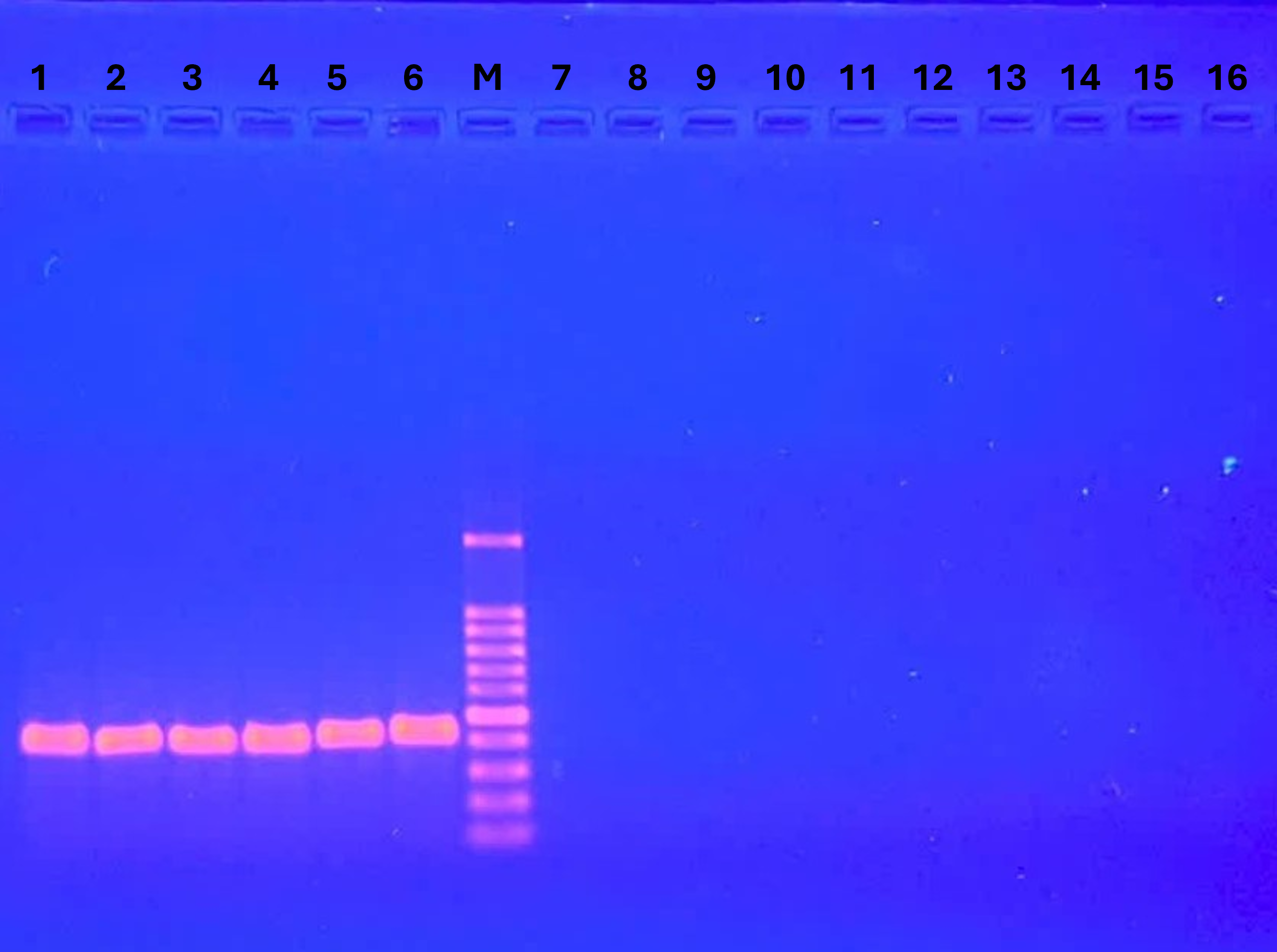


**Supplemental Figure 1. Specificity of PCR amplification for *E. tarda* using ETF and ETR primers.** PCR amplification was performed with ETF and ETR primers to assess assay specificity across multiple bacterial species. Lanes 1–6 contain representative *E. tarda* isolates from each MLST-defined clonal lineage (E24.1, E24.5, E24.7, E24.9, E24.14, and E24.16, respectively). Lanes 7–12 contain *Edwardsiella ictaluri* strains (reference strain E1 [CM129897.2] and five laboratory strains). Lanes 13–14 contain *Aeromonas dhakensis* strains (two isolates), and lanes 15–16 contain *Escherichia coli* strains (two isolates). Lane M contains a 100 bp DNA ladder (HyperLadder 100 bp).

**Supplemental Figure 2**. **Dose-dependent survival curves for 15 *E. tarda* strains in experimentally infected striped catfish.** Kaplan-Meier survival plots show the percent survival of striped catfish challenged with each of the 15 *E. tarda* strains at four bacterial concentrations: 10³, 10⁴, 10⁵, and 10⁶ CFU/mL (n = 60 fish per dose group). Each panel represents catfish-challenged studies with a single *E. tarda* strain, with survival monitored over a 14-day period post-infection. Catfish in the sham control uninfected group (orange line) showed no mortality, while fish infected with *E. tarda* developed acute bacillary necrosis of Pangasius with a clear dose-dependent mortality pattern.


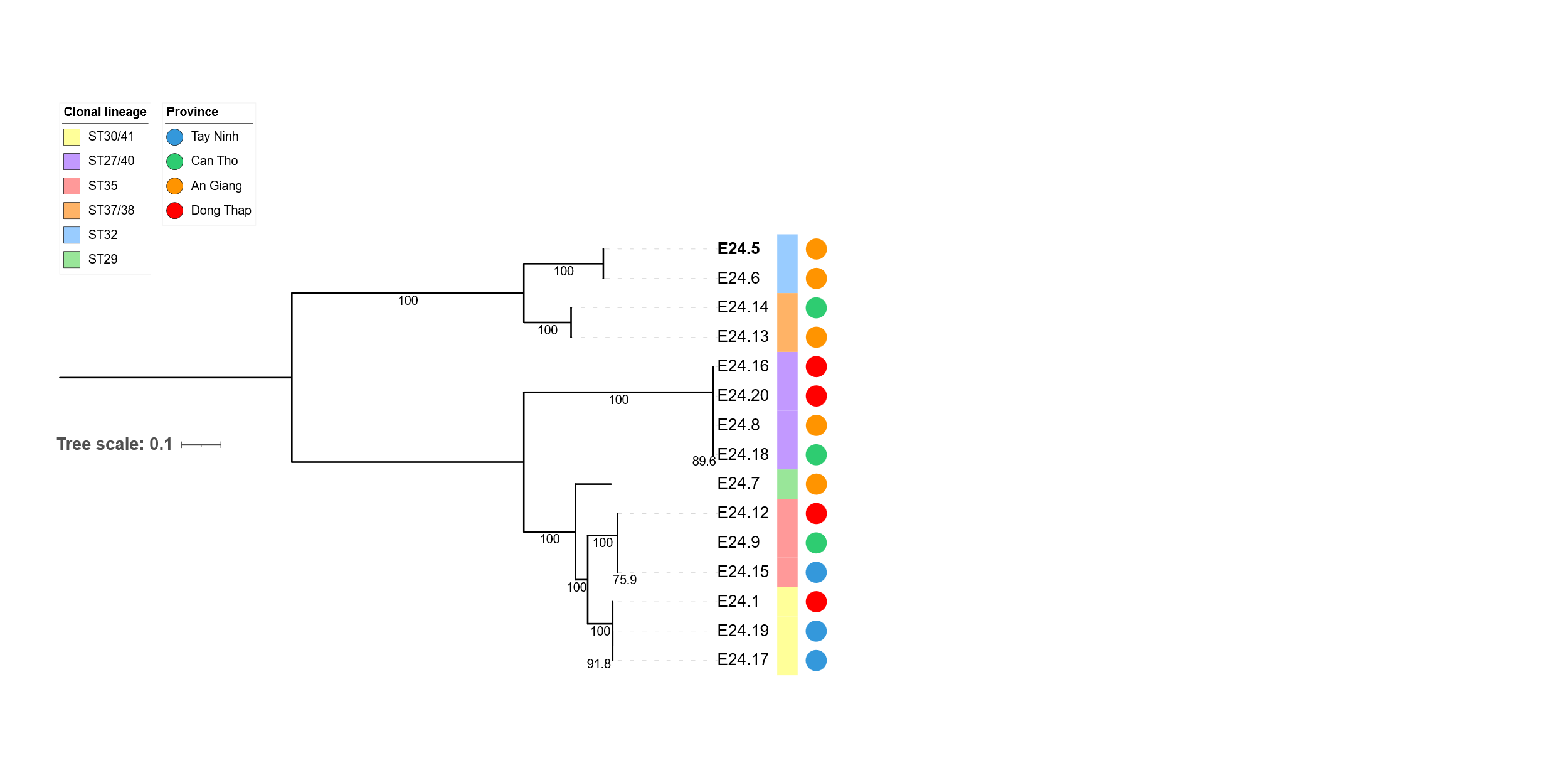


**Supplemental Figure 3. Core-genome SNP phylogeny of *E. tarda* strains.** A maximum-likelihood phylogenetic tree was constructed based on single-nucleotide polymorphisms (SNPs) identified in the core genome shared by all 15 strains, using strain E24.5 as the reference. Branch lengths are proportional to the number of substitutions per site, with the scale bar shown at the top. Bootstrap support values (percentage), calculated from 1,000 replicates, are indicated at internal nodes. Colored vertical bars adjacent to strain identifiers denote multilocus sequence types, while colored circles indicate the province of origin (Tây Ninh, Cần Thơ, An Giang, or Đồng Tháp).


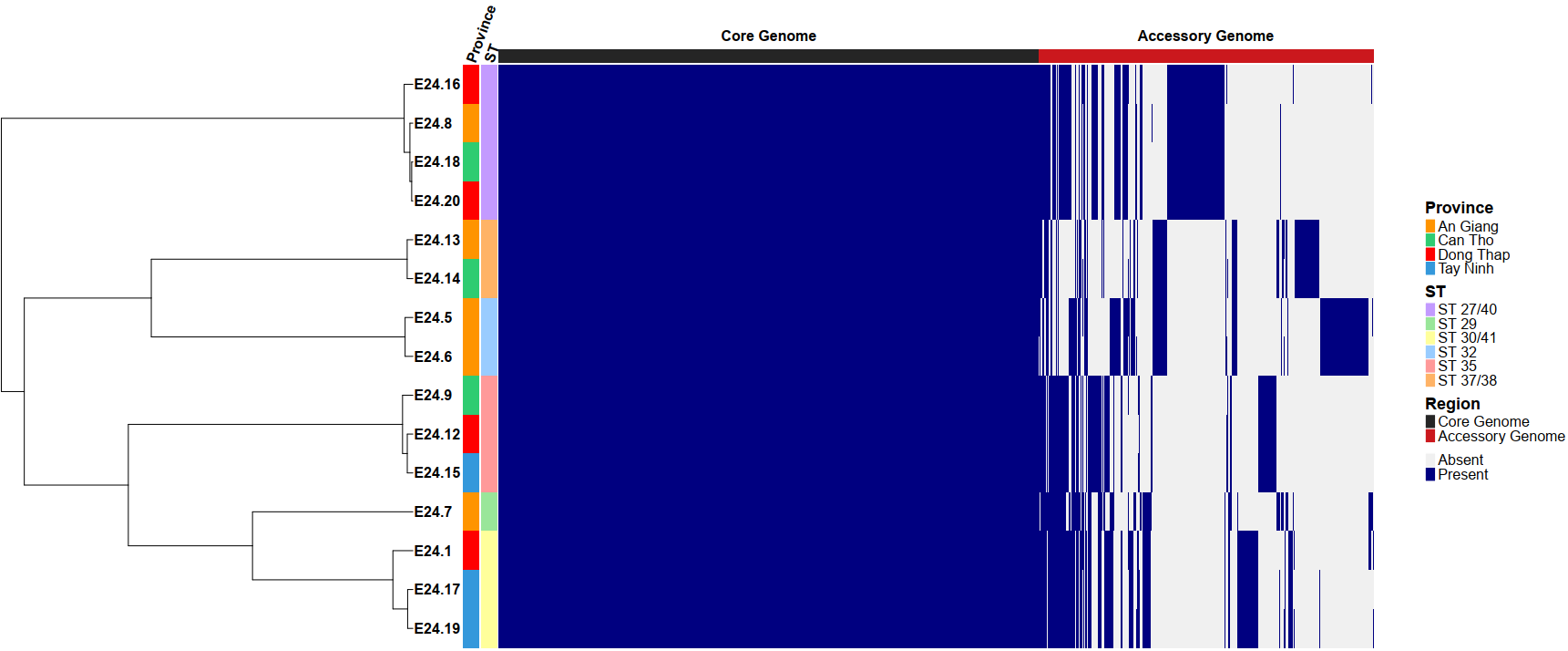


**Supplemental Figure 4. Comparative pangenome analysis of 15 *E. tarda* strains from Vietnam.** A maximum-likelihood phylogenetic tree (left) was constructed using binary presence/ absence profiles of accessory genes. Each row represents a distinct *E. tarda* strain. Colored blocks adjacent to the tree indicate multilocus sequence type (MLST), clonal complex, and province of origin. The heatmap (right) displays the gene presence/absence matrix, with core genome genes to the left (black bar) and accessory genome genes to the right (red bar). Blue blocks indicate gene presence and white blocks indicate absence.


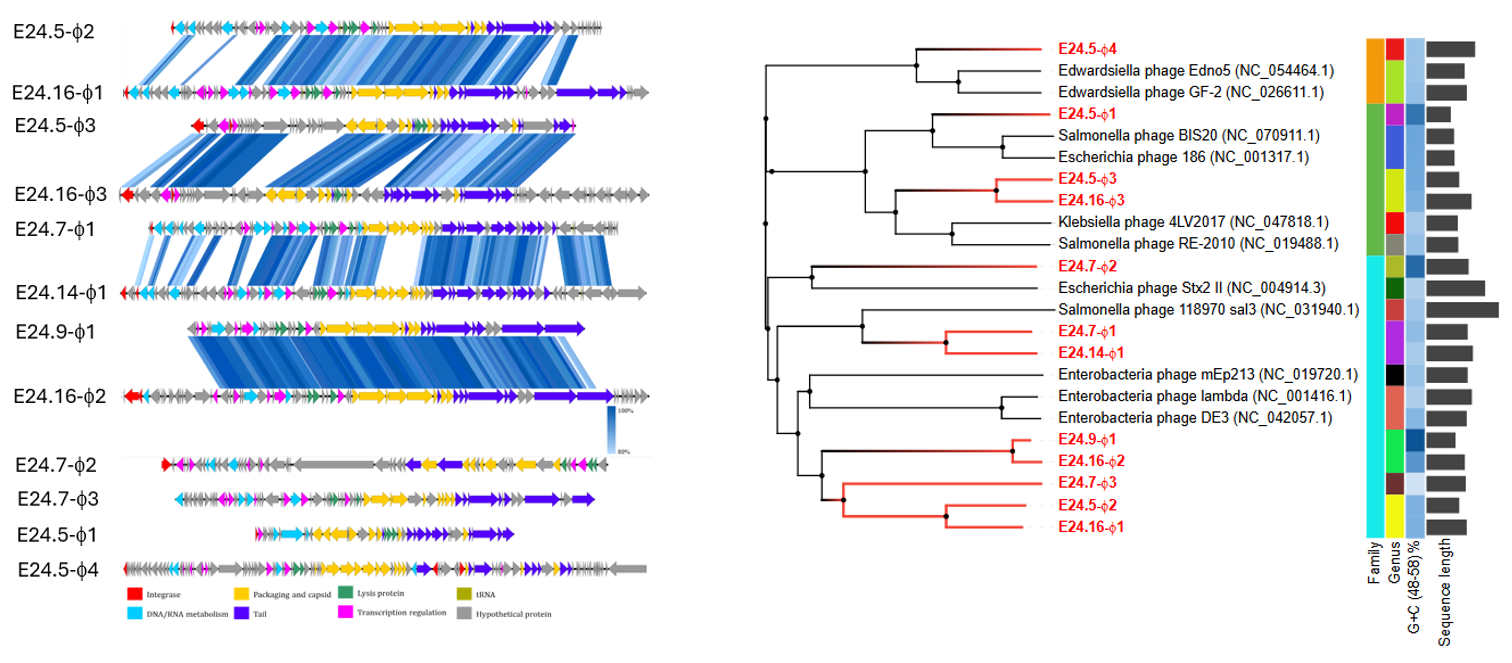
**Supplemental Figure 5. Comparative analysis of 12 complete prophages identified in *E. tarda* strains.** (**A**) Genomic synteny of the 12 prophages identified among 15 *E. tarda* strains, shown as linear representations. Coding sequences are displayed as arrows and color-coded by predicted function. Sequence similarity between prophages was assessed using the tBLASTx algorithm and is depicted by blue shading, with intensity corresponding to nucleotide identity (80–100%). (**B**) Phylogenetic tree of the 12 *E. tarda* prophages, constructed alongside reference phages from the NCBI viral RefSeq database. Prophage names from this study are highlighted in red.


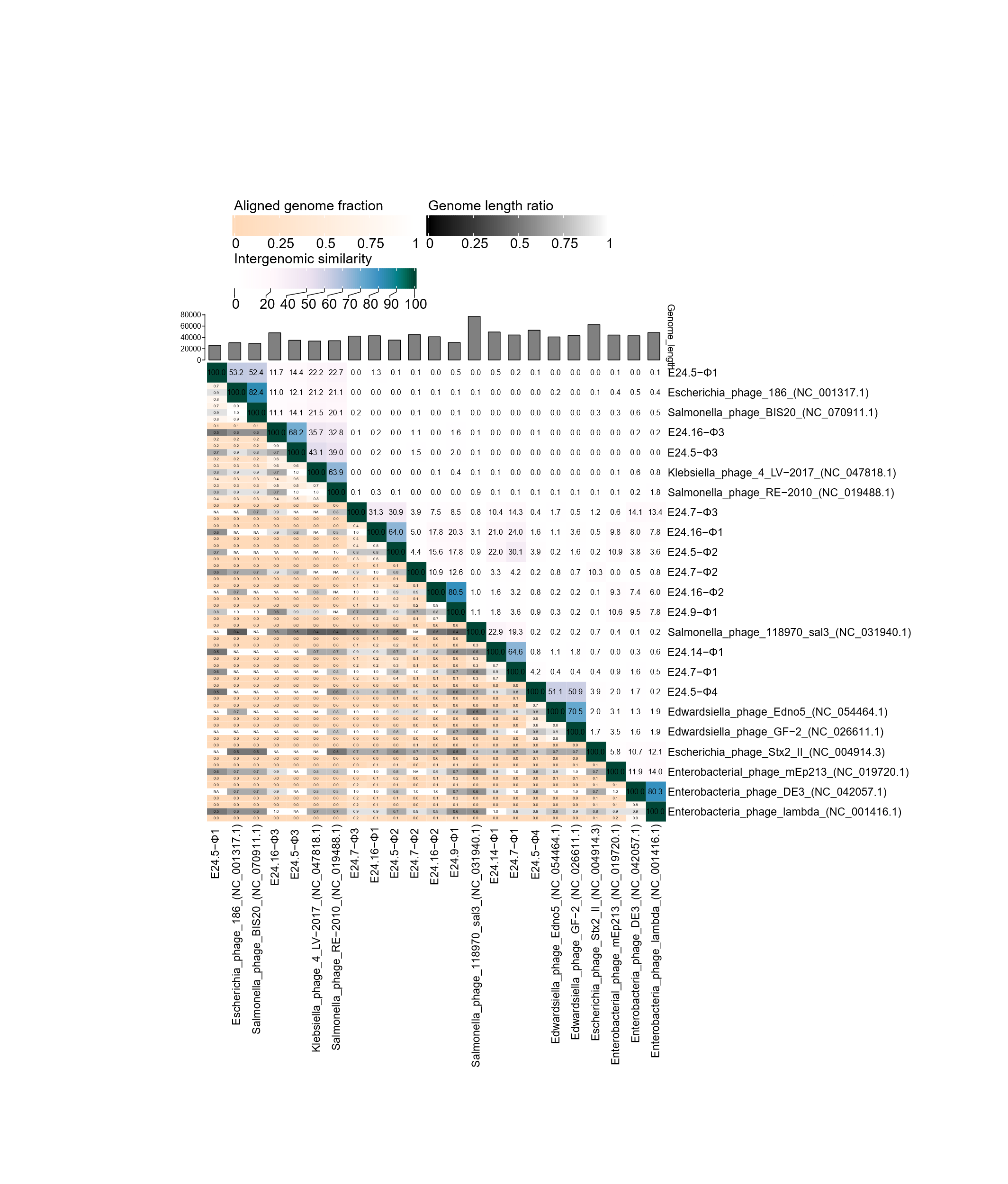


**Supplemental Figure 6. Intergenomic similarity of *E. tarda* prophages and reference phage genomes.** Heatmap shows pairwise nucleotide-based intergenomic similarity among 12 *Edwardsiella tarda* prophages and selected reference phage genomes from the NCBI viral RefSeq database. Central numerical values and the green color scale indicate percent intergenomic similarity. The orange gradient denotes the fraction of the genome aligned between each pairwise comparison, while the gray bars represent the genome length ratio. Intergenomic similarity thresholds recommended by the International Committee on Taxonomy of Viruses (ICTV) were used to support assignment of prophages to putative families and genera.

**REFERENCES**

1. **Dang THO, Xuan TTT, Duyen LTM, Le NP, Hoang HA.** 2021. Protective efficacy of phage PVN02 against haemorrhagic septicaemia in striped catfish Pangasianodon hypophthalmus via oral administration. J Fish Dis **44:**1255-1263.

2. **Krueger F.** 2015. Trim Galore!: A wrapper around Cutadapt and FastQC to consistently apply adapter and quality trimming to FastQ files, with extra functionality for RRBS data. Babraham Institute.

3. **Wick RR, Judd LM, Gorrie CL, Holt KE.** 2017. Unicycler: Resolving bacterial genome assemblies from short and long sequencing reads. PLoS Comput Biol **13:**e1005595.

4. **Walker BJ, Abeel T, Shea T, Priest M, Abouelliel A, Sakthikumar S, Cuomo CA, Zeng Q, Wortman J, Young SK, Earl AM.** 2014. Pilon: an integrated tool for comprehensive microbial variant detection and genome assembly improvement. PLoS One **9:**e112963.

5. **Wick RR, Menzel P.** Filtlong. Available online: githubcom/rrwick/Filtlong (accessed on 15/05/2025).

6. **Vaser R, Sovic I, Nagarajan N, Sikic M.** 2017. Fast and accurate de novo genome assembly from long uncorrected reads. Genome Res **27:**737-746.

7. **Olson RD, Assaf R, Brettin T, Conrad N, Cucinell C, Davis JJ, Dempsey DM, Dickerman A, Dietrich EM, Kenyon RW, Kuscuoglu M, Lefkowitz EJ, Lu J, Machi D, Macken C, Mao C, Niewiadomska A, Nguyen M, Olsen GJ, Overbeek JC, Parrello B, Parrello V, Porter JS, Pusch GD, Shukla M, Singh I, Stewart L, Tan G, Thomas C, VanOeffelen M, Vonstein V, Wallace ZS, Warren AS, Wattam AR, Xia F, Yoo H, Zhang Y, Zmasek CM, Scheuermann RH, Stevens RL.** 2023. Introducing the Bacterial and Viral Bioinformatics Resource Center (BV-BRC): a resource combining PATRIC, IRD and ViPR. Nucleic Acids Res **51:**D678-D689.

8. **Langmead B, Salzberg SL.** 2012. Fast gapped-read alignment with Bowtie 2. Nat Methods **9:**357-359.

9. **Li H.** 2018. Minimap2: pairwise alignment for nucleotide sequences. Bioinformatics **34:**3094-3100.

10. **Jolley KA, Bray JE, Maiden MCJ.** 2018. Open-access bacterial population genomics: BIGSdb software, the PubMLST.org website and their applications. Wellcome Open Res **3:**124.

11. **Bujan N, Balboa S, J LR, A ET, Magarinos B.** 2018. Population genetic and evolution analysis of controversial genus Edwardsiella by multilocus sequence typing. Mol Phylogenet Evol **127:**513-521.

12. **Khachatryan L, Kraakman MEM, Bernards AT, Laros JFJ.** 2019. BacTag - a pipeline for fast and accurate gene and allele typing in bacterial sequencing data based on database preprocessing. BMC Genomics **20:**338.

13. **Grant JR, Enns E, Marinier E, Mandal A, Herman EK, Chen CY, Graham M, Van Domselaar G, Stothard P.** 2023. Proksee: in-depth characterization and visualization of bacterial genomes. Nucleic Acids Res **51:**W484-W492.

14. **Wishart DS, Han S, Saha S, Oler E, Peters H, Grant JR, Stothard P, Gautam V.** 2023. PHASTEST: faster than PHASTER, better than PHAST. Nucleic Acids Res **51:**W443-W450.

15. **Page AJ, Cummins CA, Hunt M, Wong VK, Reuter S, Holden MT, Fookes M, Falush D, Keane JA, Parkhill J.** 2015. Roary: rapid large-scale prokaryote pan genome analysis. Bioinformatics **31:**3691-3693.

16. **Gu Z, Eils R, Schlesner M.** 2016. Complex heatmaps reveal patterns and correlations in multidimensional genomic data. Bioinformatics **32:**2847-2849.

17. **CLSI.** 2018. Methods for dilution antimicrobial susceptibility tests for bacteria that grow aerobically; approved standard, 10th ed. M07-A9. Clinical and Laboratory Standards Institute, Wayne, PA.

18. **Evenhuis JP, Welch T, Booth N.** 2013. Transferable green fluorescence-tagged pEI2 in Edwardsiella ictaluri and preliminary investigation of its effects on virulence. Dis Aquat Organ **105:**75-79.
